# Macrophages as central mediators of sympathetic neuro-immune interplay in autoimmunity

**DOI:** 10.3389/fimmu.2025.1654382

**Published:** 2025-12-16

**Authors:** Elke M. Muntjewerff, Vijay S. Josyula, Franziska Lehmair, Gustaf Christoffersson

**Affiliations:** 1 Department of Medical Cell Biology, Uppsala University, Uppsala, Sweden; 2 Science for Life Laboratory, Uppsala University, Uppsala, Sweden

**Keywords:** inflammation, neuro-immune interactions, macrophages, neurotransmitters, neuropeptides

## Abstract

The immune and nervous systems share common signaling mediators that enable neuroimmune communication, particularly at barrier tissues such as the gut, skin, and lungs. Emerging evidence implicates disturbances in this neuroimmune crosstalk—especially between sympathetic nerves and macrophages—in the pathogenesis of autoimmune diseases. This review highlights the role of sympathetic nerve–macrophage communication in maintaining tissue homeostasis and how its disruption contributes to autoimmune inflammation. Loss of sympathetic innervation, altered catecholamine levels, and imbalance between sympathetic and sensory nerves have been observed in rheumatoid arthritis, type 1 diabetes, sarcoidosis, inflammatory bowel disease, and alopecia areata. Mechanistically, macrophages interact with sympathetic neurons via β_2_-adrenergic receptor signaling or may act as norepinephrine sinks, modulating local inflammatory responses. Reduced norepinephrine availability and impaired adrenergic signaling correlate with increased cytokine production and tissue damage. Restoring neuroimmune communication through β-adrenergic modulation, macrophage-targeted therapies, or neuromodulation devices shows promise in preclinical and early clinical studies. We propose that targeting sympathetic neuro-immune interactions offers a novel, personalized therapeutic avenue for autoimmune disorders, emphasizing the need for deeper mechanistic understanding of nerve–macrophage dynamics across disease contexts.

## 1 Introduction

Despite recent advances in understanding and leveraging immune regulatory processes in autoimmune diseases, we still do not fully understand how these chronic disorders are regulated. In an evolutionary perspective, the nervous and the immune system share common mediators and receptors for crosstalk between themselves (1). This crosstalk has recently been highlighted to be of importance at barrier sites where immune cells and nerves are often found juxtaposed such as the gut, skin, and lungs. This closeness of neurons and immune cells allows for neuro-immune communication systems, which provides efficient sensing and effector mechanisms against intruders (2). Additionally, the growing field of neuroimmunology has taught us that immune cells not only respond to internal and external threats but also respond to signals from the nervous system. Early examples of research and insights in this field are how the hypothalamic-pituitary-adrenal axis controls immunity (3), the fever response (4), liver/gut-brain-axis controlling immune cell infiltration (5, 6), and central perception of inflammation (7).

The autonomic and somatosensory nervous systems have a wide coverage of our tissues, and respond to changes with much greater speed than immune cells. The interactions of these two systems in homeostasis thus become very relevant in homeostatic processes that require rapid action through nerves, and efficient effector functions through immune cells. An example of integration between the sensory and autonomic systems is the cholinergic “anti-inflammatory reflex”. Here, vagal afferent sensory neurons at inflamed sites will activate a brainstem circuit to emit vagal efferent signals to decrease cytokine production (8). Local reflex arcs are also present within individual neurons, where nociceptor neurons can respond to changes in the microenvironment at one axon that triggers release of neuropeptides in another axon to target for example immune cells in the vicinity (9). Increasing knowledge on the control of homeostasis that is balanced via sympathetic neuro-immune interplay opens new questions on how disturbances in this system affect onset of autoimmunity. For example recent findings showed that the sympathetic nervous system is involved in controlling the emergence of hematopoietic stem cells from the bone marrow (10), inducing the circadian rhythm which controls lymphocyte infiltration into tissues and lymphocyte egress from lymph nodes (11, 12), regulating T cell exhaustion via β1 adrenergic receptors (13). The involvement of sympathetic neural input on the control of immune processes leading up to autoimmune disease has been implied for a long time as an explanation for the patchy hair loss in alopecia, and the lobular inflammation seen in the T1D pancreas (14–16).

Among immune cells that potentially could respond to neuronal signals, macrophages consist a subset that is by nature primed to respond to changes in their microenvironment, and have a presence in most tissues as tissue resident macrophages including Langerhans cells in the skin. As such, they have a close connection to their organ of residence, and possess effector functions to revert potential changes and threats to homeostasis. Neurons add another layer of control to these processes.

To highlight the role of the neuro-immune field in autoimmunity, we aim to connect the observations from relevant local nerve-macrophage interactions to various autoimmune disorders. In this review, we compare the emerging data of nerve/macrophage ratios in tissues, neuronal activity, and catecholamine presence in homeostasis and several autoimmune diseases (e.g., rheumatoid arthritis (RA), inflammatory bowel disease (IBD), type 1 diabetes (T1D), sarcoidosis, and alopecia areata). We point to observations that affect target organ homeostasis such as 1) the loss of sympathetic nerves at target organs during disease 2) increase in inflammatory cytokines and chemokines 3) reduced local norepinephrine (NE) levels, and 4) disturbed communication between sympathetic nerves and macrophages via adrenergic signalling and NE sink in the affected organs. Currently, new treatments and clinical trials interfering in sympathetic nerve activity and inflammation via drugs and/or neurostimulators are surging. Therefore, we also touch up on the development of current treatments and future clinical implications to stimulate neurogenesis and/or local macrophages via drugs or neurostimulation treatment resulting in release of anti-inflammatory and neuron aiding factors. Altogether, we aim to provide a review that connects knowledge from the sympathetic neuro-macrophage field with observations in autoimmunity to highlight an important angle of research in diseases where we still are lacking cures and insights in etiology.

## 2 Main text

### 2.1 Sympathetic nerve loss drives autoimmune related inflammation

#### 2.1.1 In autoimmune diseases, inflammation correlates with sympathetic nerve loss

The presence of sympathetic nerves seems important to maintain tissue homeostasis since the loss of them is reported for various autoimmune diseases including RA (17, 18), T1D (14, 15), sarcoidosis (19, 20), IBD (21) and alopecia areata (22). Already in 1996, a clinical study using radioactive tracers ^201^Tl (Tl-thallous chloride) and ^123^I-MIBG (meta-iodobenzylguanidine), myocardial scintigraphy indicated regional disruption of the adrenergic nervous system in sarcoid heart disease (20). Sarcoidosis is an autoimmune-related disease characterized by granulomatous inflammation of the lungs, skin, lymph nodes, heart, liver and eyes. In ~20% of the cases, the CNS or peripheral nervous system are affected as well, this is called neurosarcoidosis (19). During progression of T1D, the β-cells in the pancreatic islets are destroyed in an autoimmune process resulting in lifelong dependence of exogenous insulin (23). Interestingly, at the start of the beta cell loss in pre-diabetic mice, the sympathetic nerves (TH^+^, Neuropeptide Y^+^) in the islets are no longer present (14, 15). This has been found also in the human pancreas in both recent-onset T1D and in at-risk individuals (autoantibody positive).

Although most autoimmune diseases display as loss of sympathetic nerves, in IBD this is debatable. One study reported a loss of sympathetic nerves (21), while other studies have shown an increase of both sympathetic and sensory nerves mainly located around the mesenteric vessels (24) and in the mesenteric plexus (25). The contradictory results might be due to use of different material from colitis or Crohn´s patients in various states of disease (inflamed or non-inflamed intestine). A larger cohort of patients, where tissue collection is synchronized to a similar disease state would be necessary to define the nerve network changes in IBD.

The loss of sympathetic nerves is sometimes accompanied by an increase in sensory nerves, as observed in RA patients, where characterisation of the synovial tissue showed a reduction in sympathetic nerves (TH^+^) and an increase in sensory nerves (substance P^+^). Only the synovial tissue of RA patients displayed a disturbance in nerve balance and not the synovial tissue of osteoarthritis patients, which suffer from non-auto-immune related breakdown of cartilage in the joints (17, 18). Also, the activation of sensory and sympathetic nerves plays a role in autoimmune disease development. The activation of soleus sensory nerves in the muscle resulted in sympathetic nerve activation and CCL20 expression resulting in IL-6 release in the fifth lumbar cord in a mouse model of multiple sclerosis (MS), where, impairment of the sensory nerves resulted in reduced local IL-6 dependent chemokine expression (26). In line with this, canines with induced acute ischemic stroke showed less occurrence of ventricular tachyarrhythmias, decreased macrophage infiltration (iNOS^+^, CD68^+^, MCP-1^+^) and lower TNF-α levels after blocking the sensory nerves via left stellate ganglion ablation (27). This indicates that the balance between sensory and sympathetic activation is important to maintain local organ homeostasis. In summary, the loss of sympathetic nerves and increase of sensory nerves is related to only auto-immune based inflammation in these cases. Additionally, the activation of sensory nerves can in turn result in the increased release of inflammatory factors via the activation of sympathetic nerves possibly contributing to the acceleration of autoimmune disease in the target organs.

#### 2.1.2 Effect of sympathetic nerve loss on autoimmune-related inflammation

The loss of sympathetic nerves seems to be connected to autoimmune-related inflammation in RA and T1D. For example, in synovial fluid from RA patients only the sympathetic nerve reduction correlated with the inflammation index (18). Mounting data show that sympathetic nerves seem to control local homeostasis, therefore the local loss of these nerves could alter local immune responses. In line with this, electrical vagus nerve stimulation in wildtype mice decreased tumor-necrosis factor-α (TNF-α) in the blood and production from isolated macrophages. However, if the researchers stimulated nicotinic acetylcholine receptor α7 (nAChRα7)- deficient mice, TNF-α levels were no longer decreased in both the blood and as produced by isolated macrophages (28). This suggests that, the sympathetic signalling, via nAChRα7, is indeed important to control the immune response via inhibition of TNF-α (28). To place these findings in a more autoimmune disease perspective, pancreatic sympathetic nerve electrical stimulation via microelectrodes in non-obese diabetic (NOD) mice also resulted in inhibited disease progression with lower glycemia levels, less beta cell loss and severe immune cell infiltration in pancreatic islets when compared to NOD mice with sham treatment (29). To simulate local destruction of dopaminergic and noradrenergic nerves, researchers use the neurotoxin 6-hydroxydopamine (6-OHDA) treatment. In the case of hair loss, as observed in alopecia areata patients, chemical sympathectomy via administration of 6-OHDA) inhibits hair follicle growth and keratinocyte proliferation when compared to untreated mice of the same model (30). Additionally, treatment of new-born mice with 6-OHDA results in hairless skin at the injection site that recovers 20 days after the treatment (31). The immune attack of hair follicles resulting in hair loss for alopecia areata patients might thus be driven by the loss of sympathetic nerves.

Moreover, the loss of sympathetic innervation by 6-OHDA treatment or surgical sympathectomy by cutting the superior mesenteric nerve in Rag1-/- mice, who lack T and B cells, resulted in dropping NE levels, an increase in colon pro-inflammatory cytokine levels (IL-1β, IL-6 and IL-10) and intestinal histological features that correspond with colitis including a decrease in goblet cells (32). Again, highlighting the importance of sympathetic signalling for organ homeostasis.

In contrast, chemical blockage of sympathetic signalling, via 6-OHDA treatment, halts T1D onset in an autoimmune diabetes mouse model (RIP-LCMV-GP) (15). This suggests that, interfering with sympathetic nerve signals to the pancreas could prevent patients from developing T1D. Also physical denervation, by cutting the efferent tyrosine hydroxylase–positive nerve bundle innervating the pancreas at the superior mesenteric artery, resulted in less progression towards diabetes (15%) when compared to the mice that underwent sham surgery or vehicle treatment (100%) (15). In line with these findings, T1D patients show more nerve network and more immune infiltration (including macrophages, neutrophils and T cells) in the pancreas during T1D onset (33). The study doesn´t define if the observed increase in nerve network is based on sympathetic or sensory nerves. Thus, loss of the pancreatic sympathetic nerves before the onset of autoimmune diabetes protects the pancreas from the autoimmune attack, possible due to a protective effect on the pancreatic immune homeostasis.

Altogether, this indicates that the connection between the local immune system and sympathetic nerves plays an important role in the development of autoimmune disease. In summary, tissues of patients with autoimmune disease display specific sympathetic nerve loss (14, 15, 17–20), which might even coincide with an increase in sensory nerves. Although the cause of the sympathetic nerve loss remains unknown, it seems to drive the inflammation in autoimmune diseases since it correlates to autoimmune related inflammation in RA, alopecia areata and T1D (15, 18, 29, 30). Additionally, interference in the local homeostasis by blockage or stimulation of the sympathetic nerves changes disease outcomes in mouse models (15, 29–31).

### 2.2 Underlying mechanisms during autoimmune disease onset leading to disruptions in the sympathetic nerve-macrophage communication

#### 2.2.1 Inflammation is related to reduced NE levels

In an attempt to reflect NE metabolism in lungs during sarcoidosis, I-^123^ meta-iodobenzylguanidine (MIGB) washouts were performed (34). Here the decreased lung washout of I-^123^ MIGB suggests that the sympathetic autonomic nerve dysfunction is linked to lung inflammation (serum measured IL-2 and C-reactive protein (CRP)) (34). To place this in a broader perspective, similar results have been found for type 2 diabetes patients and patients with high altitude pulmonary edema (35, 36).

**Figure 1:**
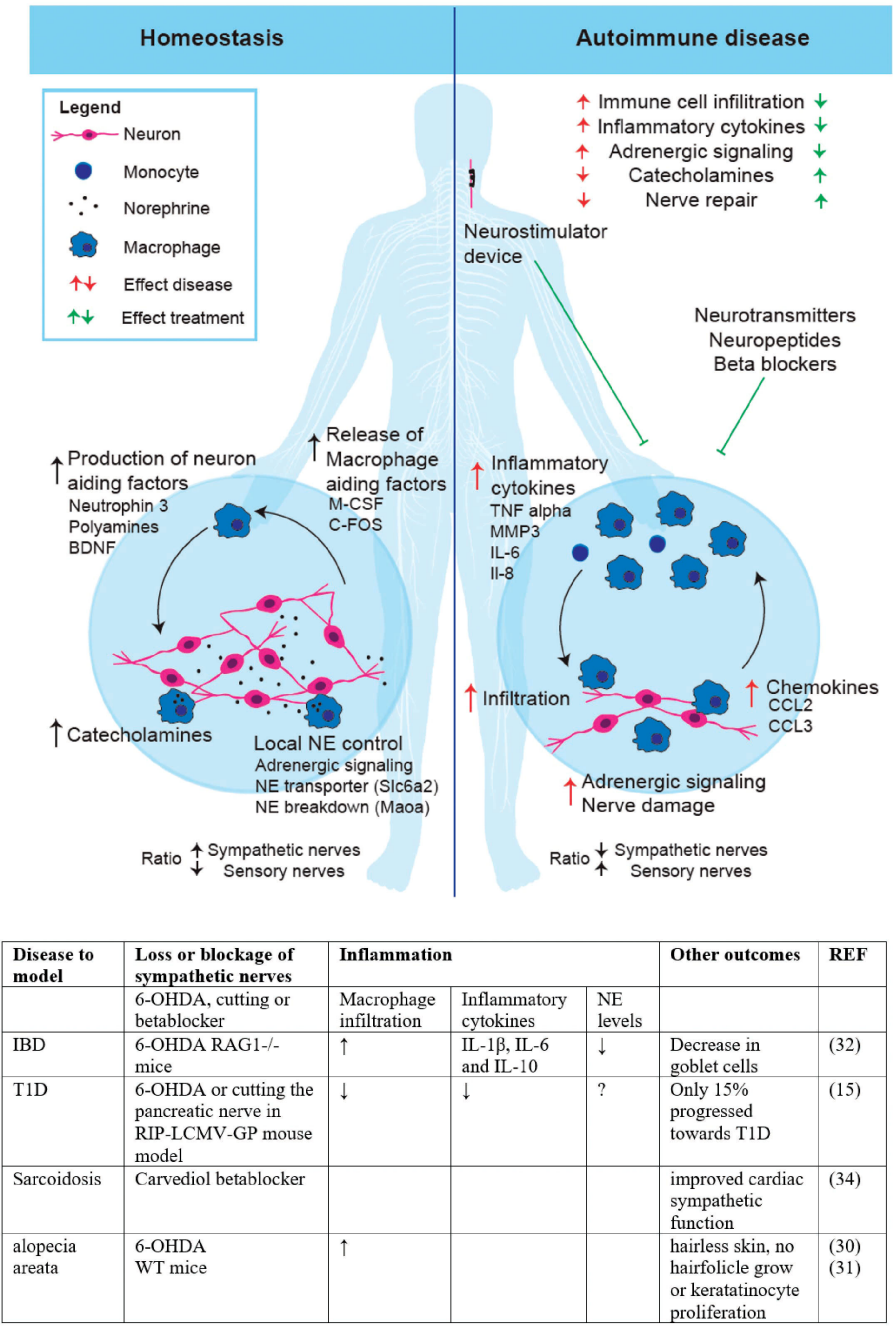
Nerve immune interactions in Homeostasis and autoimmune disease. Homeostasis (Left): In a healthy situation neurons and macrophages aide each other via the release of stimulating factors. Neurons release Macrophage stimulating factor (M-CSF) and Fos proto-oncogene (C-FOS), while macrophages release Neutrophin 3, Polyamines and Brain derived neurotrophic factor (BDNF). There are catecholamines in the tissue, which can be locally fine-tuned via the macrophages using adrenergic signaling, uptake via NE transporter Solute Carrier Family 6 Member 2 (SLC6a2) or breakdown via Monoamine oxidase A (Maoa). As a result, the ratio between sympathetic nerves and sensory nerves is in balance. Autoimmune disease (Right) In autoimmune disease, tissues suffer from immune cell infiltration due to high levels of chemokines (CCL2, CCL3) and inflammatory cytokines (e.g., Tumor Necrosis Factor alpha (TNF alpha), matrix metalloproteinase-3 (MMP3), Interleukin (IL) IL-6, IL-8). Patients with autoimmune disease display low levels of catecholamines and nerve damage (mostly sympathetic nerves) probably due to increased adrenergic signalling and tissue damage. Interfering in this autoinflammatory state seems possible using neuromodulation (neurostimulatory devices) or nerve modulating drugs including neurotransmitter (e.g., NE), neuropeptides (e.g., Neuropeptide Y, Catestatin) or beta blockers. (created with BioRender.com). (Table) Shows the summarized effects of sympathetic nerve blockage studies. The studies are sorted per disease that is intended to model by blockage of the nerves via neurotoxin 6-hydroxydopamine (6-OHDA), sympathetic nerve cutting or betablocker treatment. Inflammatory bowel disease (IBD), Type 1 diabetes (T1D), Norepinephrine (NE), C-reactive protein (CRP), increase (↑), decrease (↓).

Mixed synoviocytes isolated from the synovial fluid around the inflamed joint of RA patients showed increased inflammatory cytokine release (IL-6, IL-8, MMP3) and reduced NE release (18). Thus, the loss of sympathetic nerves seems related to increased synovial inflammation in RA via reduced NE production and increased inflammatory cytokines, probably driving the immune influx (**Figure 1**).

#### 2.2.2 During inflammation macrophages and sympathetic nerves communicate via β-adrenergic receptor signalling

The bridge connecting the macrophages and sympathetic nerves to regulate remodelling and restoration of injured tissue seems to be β-adrenergic receptor signalling (β2AR) (37). The lack of β2AR in chimeric mice followed by surgically induced myocardial infarction resulted in 100% mortality and no macrophage (CD68^+^) infiltration (37), highlighting the importance of β2AR signalling in wound healing.

In the intestine, muscularis macrophages are situated close to sympathetic nerves and express β2AR (38). Here sympathetic neurons and macrophages have been found to work in concert during inflammation as found by c-fos-expressing neurons and release of NE, which activates the muscularis macrophages via the β2AR. This activation of β2ARs triggers the macrophages to release polyamines to protect the nerves (38, 39) (**Figure 1**). Additionally, the release of NE triggers pro-inflammatory cytokine release since treatment of U937 macrophages induced IL-6 expression and release as accessed by ELISA and qPCR. The effect seems to depend on the β2AR and NF-kβ signalling pathway since the addition of propranolol, NF-kB inhibitor PDTC, NAD(P)H oxidase inhibitor DPI or antioxidant NAC one hour before the NE stimulation decreased IL-6 mRNA expression or release (40). This response may however be dependent on the inflammatory status of the macrophages. In inflammatory, LPS stimulated murine bone marrow derived macrophages, NE treatment resulted in lower levels of pro-inflammatory cytokines (IL-6, TNF-α) and increased arginase-1 (Arg-1, anti-inflammatory marker) expression, where this effect could be reversed by treatment with the β2AR antagonist propranolol (32).

Co-cultures of enteric-associated neurons with peritoneal or RAW macrophages show that macrophage polarization plays a role in this aspect as addition of the β2AR agonist salbutamol induced anti-inflammatory marker Arg-1expression upregulation, while the β2AR antagonist butaxamine reduced Arg-1 levels (38). This indicates that β2AR signalling is necessary to activate and control the inflammatory status and response in macrophages. In line with this, during enteric infections, β2AR signalling is activated to protect neuron loss as shown in mice intragastrically exposed to Salmonella typhimurium (spiB) (38, 39, 41). As a follow-up, mice where first exposed to the less harmful bacteria Y. pseudotuberculosis before exposure to spiB, which resulted in neuroprotection as measured by a decrease in intestinal neural loss (ANA-1^+^) and improved gut motility (41). This neuroprotection again seems mediated by β2AR signaling in macrophages since macrophage depletion (via anti-CSF1R antibody treatment) resulted in major neuron loss upon exposure to spiB (41). To confirm the role of *Arg1*, the exposure to Y. pseudotuberculosis followed by spiB was repeated in *LysMΔArg*1 mice (in which *Arg-1* is conditionally deleted in myeloid cells), which again resulted in significant neuron loss (ANA-1^+^) upon exposure to the same viral load (41). Altogether, it seems that primary exposure results in disease tolerance via macrophage β2AR signaling and neuronal protection. However, to know the effects on the various types of intestinal nerves, extensive staining for sympathetic (TH^+^, neurofilament^+^) and sensory (substance P^+^, protein gene product 9.5^+^) nerves would need to be performed in the future. In the light of autoimmune disease e.g., IBD, it would be interesting to know whether only the intestinal neurons are protected based on previous viral or bacterial exposure or if these signals are carried on further in the body via the nervous system to possibly even aid in improving immune responses and neuronal resilience systemically. In general, it seems that communication between tissue resident macrophages and sympathetic neurons via β-2AR signalling is necessary to activate an inflammatory response, where blockage of the β-2AR signalling results in an anti-inflammatory response. More research is needed to define how we can fine-tune the communication between tissue resident macrophages and sympathetic nerves via β-2AR signalling in autoimmune onset.

#### 2.2.3 To maintain homeostasis, macrophages act as NE sinks to control the local environment

As discussed above, macrophages are able to directly receive information from the sympathetic neurons via β2AR signalling. These nerve associated macrophages (SAM)(CD45^+^F4/80^+^) cannot produce NE themselves, but they do express the gene encoding the NE transporter (solute carrier family 6 member 2, *SLCA2*) and the gene for metabolizing down NE (monoamine oxidase A, *MAOA*) (42). Thereby, macrophages can act as NE sinks in a process where NE is internalized through SLC6A2 channels, and eliminated using MAOA (42, 43). This process is useful to control local homeostasis, for example in adipose tissue. Here, the macrophage NE sink is used to regulate fat homeostasis by inducing lipolysis through decreased local catecholamine levels.

The NE sink provides an important targetable nerve-macrophage connection that could be used to control local processes. The inhibition of MAOA by clorgyline treatment led to increased local NE levels and increased lipolysis in aged mice (43) (**Figure 1**). and in increased intracellular NE levels in *in vitro* cultured CX3CR^+^ cells co-cultured with superior cervical ganglia (42). Also, in chronic inflammation, the macrophage NE sink plays an important role as type 2 diabetic mice (*ob/ob*) that receive bone marrow from diabetic mice with a deletion for the NE transporter (*ob/ob-Slc6a2^−^/^−^*) display increased NE levels, rescued thermogenesis and decreased weight compared to normal *ob/ob* mice (42). Altogether this indicates that nerve-associated macrophages control homeostasis via the NE sink, which might be controlled via β2 adrenergic signalling as discussed above. More research is necessary in autoimmune disease models to prove this link in the context of autoimmune diseases (e.g., T1D, RA, IBD, sarcoidosis, multiple sclerosis, and SLE) and to test the therapeutic potential of targeting macrophage-sympathetic nerve communications to maintain organ homeostasis.

## 3 Future treatments for autoimmune diseases targeting the nerve network

In most autoimmune diseases discussed above, inflammation seems to correlate to the loss of sympathetic nerves and thereby disturbed macrophage-nerve interactions (**Figure 1**). Therefore, the development of treatments interfering in neuroimmune communication via drugs and/or electronic neurostimulators is spiking (**Table 1**).

**Table 1:** Recent clinical trials related to autoimmune disease treatment.

Clinical trial number	Participants	Target of clinical trial	Used drug or device	REF
NCT03079921	T1D	α-adrenergic (neural) & β-adrenergic (hormonal) blockage	Phentolamine (α1- receptor blocker), Propranolol (β2-receptor blocker)	–
NCT03538015	T1D	Adrenergic beta blockage	Carvedilol 3.125 mg Carvedilol 2.5 mg	–
NCT02799472	RA	granulocyte-macrophage colony stimulating factor (GM-CSF) signaling pathway inhibition	Drug: GSK3196165 Drug: Placebo Drug: MTX Drug: Folic (or folinic) acid	(44)
NCT03316651	aPAP	Immune modulation via GMCSF	GM-CSF inhaling via proprietary nebulizer	–
NCT06431776	aPAP	Immune modulation via GMCSF	Molgramostim inhaling via proprietary nebulizer	–
NCT04955899	RA	splenic neurovascular bundle	Galvani System	–
NCT01569503	IBD (Crohn’s disease)	Vagus nerve stimulation (VNS)	implanted pacemaker-like device	(50) (51)
NCT02311660	CD	Vagus nerve stimulation (VNS)	implantable VNS device (Cyberonics VNS System)	–
NCT02951650	CD	Vagus nerve stimulation (VNS)	implantable VNS device (Cyberonics VNS)	–
NCT03953768	Epilepsy	Vagus nerve stimulation (VNS)	Vagal nerve stimulation (VNS)	–
NCT03863704	IBD (Crohn Disease or ulcerative colitis)	transcutaneous vagal nerve stimulation (VNS)	Transcutaneous Electrical Nerve Stimulation (TENS)	(52)
NCT03908073	IBD	transcutaneous vagal nerve stimulation (VNS)	Transcutaneous vagal nerve stimulation	(53)
NCT01552941	RA	Vagal nerve stimulation (VNS)	Cyberonics VNS System	(54)
NCT03437473	RA	Vagus Nerve Stimulation (VNS)	SetPoint Medical Neurostimulation of the Cholinergic Anti-inflammatory Pathway System	(55)
NCT04862117	RA	Vagus Nerve Stimulation (VNS)	SetPoint Medical Neurostimulation of the Cholinergic Anti-inflammatory Pathway System	–
NCT04539964	RA	VNS	SetPoint System; Drug: Conventional Synthetic DMARD	(56)
NCT01569789	Healthy participants	VNS	Vagus Nerve Stimulator	(57)
NCT00859859	RA	auricular stimulation of the vagus nerve (VNS)	oscillatory device (Brookstone)	(57)
NCT03327649	Heart failure with preserved ejection fraction (HFpEF)	transcutaneous electrical s)	low level transcutaneous vagus nerve stimulation	(58, 59)
NCT02359188	Healthy participants	Transcutaneous vagal nerve stimulation (tVNS)	VNS	–

The table displays the clinical trials discussed in the manuscript by providing the clinical trial number, the participants in the study, the target of the clinical trial, the name of the used device (if available) and publications related to the findings (if available). Autoimmune Pulmonary Alveolar Proteinosis(pPAP), rheumatoid arthritis (RA), crohn´s disease (CD), type 1 diabetes (T1D), inflammatory bowel diseases (IBD).

### 3.1 Autoimmune disease treatment by targeting macrophage stimulating factor

Autoimmune diseases are characterized by chronic inflammation. Since macrophages and sympathetic neurons aid each other, targeting the inflammatory macrophages can help to locally bring down the inflammation. A fairly new way to modulate the macrophage population during autoimmune diseases is granulocyte-macrophage colony-stimulating factor (GM-CSF). GM-CSF drives many inflammatory conditions, so reducing the GM-CSF levels in autoimmune diseases by the use of human monoclonal antibodies that inhibit GM-CSF seems promising. For patients with active RA, treatment with Otilimab for 12 weeks showed improved synovitis (44) (NCT02799472). These results look promising and no side effects were observed. However longer treatment is not sustainable since the researchers observed that the GM-CSF–chemokine (C-C motif) ligand 17 (CCL17) concentration decreased in the first weeks but returned to baseline levels after 12 weeks of treatment (44). Interestingly, for patients with autoimmune pulmonary alveolar proteinosis clinical trials are heading in the opposite direction by using GM-CSF stimulation to improve the lung inflammation in patients with autoimmune pulmonary alveolar proteinosis (aPAP) (NCT03316651, NCT06431776). It will be exciting to see the results from the upcoming phase 3 trial, where the drug Molgramostim will be administered once a day by inhaling using a specialized proprietary nebulizer (NCT06431776). Altogether, this non-invasive treatment might be helpful to bring down the inflammation for a short time frame.

### 3.2 Autoimmune disease treatment via nerve modulating drugs

As discussed above, communication to macrophages via catecholamines and neuropeptides has implications for both homeostasis and pathophysiology. Thereby, stimulating neurons directly results in release of anti-inflammatory cytokines from macrophages and stimulates the release of neuron-aiding factors (e.g. polyamines), which may have a beneficial effect on disease progression (38, 39). Besides that, interfering in the neurotransmitter levels by injection of neuropeptides (neuropeptide Y) or blockade via α- or β-beta adrenergic blockers or small molecules acting on G-protein coupled receptors (e.g., gallein, catestatin) is trialed (45–47). These days most betablockers are already FDA approved for use in the cardiovascular field, making it easier to run drug repurposing trials in participants with autoimmune diseases. A case study showed that, a sarcoidosis patient with small fiber neuropathy and cardiac sympathetic dysfunction (assessed with I-^123^ MIBG SPECT) improved cardiac sympathetic function upon treatment with the non-selective adrenergic receptor blocker carvediol (48). Not only sarcoidosis patients, but also patients with RA and SLE are at risk of cardiac complications including hypertension, infarctions and tachyarrhythmias (49). At the moment there are several T1D trials ongoing to investigate the effect of α- or β-beta adrenergic blockers on hypoglycaemia. One trial uses low dosses of carvedilol to investigate whether this can improve hypoglycemia in T1D patients (NCT03538015). Another study plans to finish at the end of 2025 and investigates if phentolamine (α1- AR blocker) or propranolol (nonselective β2-AR blocker) administered to T1D patients that received intrahepatic islet transplantation will affect sympathetic neural and hormonal (epinephrine) input on islet cell hormonal responses to insulin-induced hypoglycemia (NCT03079921). The outcome of these trials will not only show whether the beta blockers can be used as treatments for autoimmune diseases, they will hopefully also teach us more about disease mechanisms.

### 3.3 Neurostimulator devices to treat autoimmune disease

One of the challenges of using vagal stimulants is the possibility of stimulating both efferent and afferent nerves simultaneously that have conflicting effects. The solution is to block the afferent while stimulating the efferent vagus nerve. In LPS stimulated rats, efferent cervical vagus nerve stimulation in combination with blocking the afferent cervical vagus nerve using kilohertz electrical stimulation showed dampening of the immune system via reduced TNF-α expression (60). However, opposite results were found with an increased inflammatory profile when the afferent cervical vagus nerve was partially blocked (60). This could be a potential approach to design such neuromodulators for immune dampening and eventual reduction in autoimmune diseases.

The use of neuromodulation via electrical stimulation of the vagus nerve and/or spinal cord is an emerging clinical alternative to control pain, inflammation and to stimulate nerve repair (61, 62). Recently, a small trial with 16 patients showed that direct sciatic nerve stimulation can be used to control leg pain in complex regional pain syndrome (63). These beneficial effects seem to originate from a change in immune cell accumulation and an increase in epinephrine, NE and neuropeptide Y concentrations as observed in the liver, popliteal and axillary lymph nodes of rats after direct sciatic nerve stimulation (0.5 mA at 20 Hz pulsing frequency) (64). Although the effects seem promising, this temporary direct stimulation of the sciatic nerve is an invasive procedure and therefore not immediately ready for routine clinical use.

To be able to provide pulses for a longer time period, an alternative is to implant a vagus nerve-stimulating device (also called pulse generators). As the name indicates, these devices generate controlled pulses of various intensities over time (62). Most experience from the use of these devices originally comes from the cardiovascular field, where patients with chronic heart failure need an implanted vagus nerve-stimulating device, such as the BioControl Cardiofit system to optimise the heart rhythm (65, 66). Testing these implantable devices in animal models for autoimmune diseases showed that low pulses over a longer time period could improve the inflammatory state and autoimmune progression in T1D (29), IBD (50) and RA (67).

Based on these results, researchers and companies are currently testing implantable neurostimulator devices in patients with various autoimmune diseases. For IBD, neuromodulation seems to reduce the inflammatory response in small clinical trials using surgically implanted devices clipped around the vagus nerve in the neck (NCT03953768) and non-invasive transcutaneous vagal nerve stimulation (NCT03863704, NCT03908073). Excitingly, the neurostimulator devices are getting smaller and smaller due to improved battery technology that in some cases even can be recharged wirelessly after implantation by wearing a bracelet charger once a week. The advantage of this is that the placement is less invasive for the patient.

For CD patients the implantation of a vagus nerve stimulatory device in a six months clinical trial resulted in restored vagal tone and remission as confirmed by biomarkers, endoscopy and clinical assessment (68, 69)(NCT01569503, NCT02311660). Moreover, in RA patients implantation of the Cyberonics Vagus Nerve Stimulation device significantly inhibited TNF-α production for up to 84 days in combination with stable low dose prednisone treatment. In line with this, RA disease severity, as measured by standardized clinical composite scores, improved significantly compared to RA patients on standard treatment (54, 68, 70) (NCT02951650). Cyberonics is now Liva Nova, which currently focuses on Vagus Nerve Stimulation Therapy™ to prevent seizures in drug-resistant patients with epilepsy (NCT04539964).

Recently, the MicroRegulator system SetPoint Medical received FDA approval for its neuroimmune modulation therapy for RA after extensive testing in clinical trials (NCT01552941; NCT03437473; NCT04862117). This is the first approved device to treat RA patients using neurostimulation opening up existing possibilities for the use of neurostimulator devices in personalized medicine.

Another way to shift from a pro- to an anti-inflammatory state is to interfere in chronic inflammation via stimulation of the splenic nerve as shown in pigs (71). This splenic nerve stimulating device from Galvani Bioelectronics is surgically inserted. The implant could be kept small, as it can be externally charged by wearing a belt powering the device. The device looks promising and is currently in a trial for RA patients to test its effect on chemokine/cytokine release in humans (NCT04955899). For shorter treatments, researchers are developing bioresorbable nerve stimulators to make it less invasive for the patient since the device doesn’t have to be surgically removed after the treatment period (72).

Besides implantable devices, there are now also portable devices, such as transcutaneous vagus nerve stimulation via the ear and the Portable Neuromodulation Stimulator (PoNS™) that can be placed on the tongue (73). However, the mechanism of action or possible effects on the nerve-immune interactions remain unknown. A more convenient way seems to be transcutaneous vagus nerve stimulation. Currently, there are two variants available; transcutaneous cervical vagus nerve stimulation with a device around the neck (74) or a small removable device in the left ear that stimulates the afferent auricular branch of the vagus nerve (75). In healthy individuals, vibrotactile at the cymba concha stimulation of 4 hours resulted in decreased inflammatory cytokines (TNF-α, IL-6, IL-1β) levels in the plasma, whereas RA patients show reduced disease activity scores (DAS28-CRP) after 2–7 days of cymba concha stimulation (57) (NCT01569789, NCT00859859). Also, patients with heart failure showed reduced inflammatory cytokines (TNF-α, IL-8) in the plasma and improved global longitudinal strain after 3 months transcutaneous vagus nerve stimulation when compared to controls patients with sham stimulation (59) (NCT03327649). Regarding the gut, transcutaneous auricular vagus nerve stimulation seems to affect gastric motility where stimulation of 25Hz resulted in higher amplitudes of peristaltic waves measured using real-time MRI in humans (76) (NCT02359188). Although more research is needed to test the effects of transcutaneous vagus nerve stimulation in autoimmune diseases, these removable non-invasive devices might be very useful in the future.

Thus, in the future selective small molecules acting on GPCRs, neuropeptides, α- or β-beta adrenergic blockers, removable or bioresorbable neurostimulator devices could be very helpful to personalize treatment of patients with various autoimmune diseases.

## 4 Concluding remarks

As discussed in this review, sympathetic nerve-macrophage communication is important for tissue homeostasis. By these systems communicating, they fight infections and restore local tissue homeostasis. Disruption of this communication might even drive the autoimmune disease progression. More research is necessary to understand the role of the changes in nerve-immune communication and the shifting innervation from sympathetic to sensory in autoimmune disease progression. Their roles may be different for immune diseases since they have different ´target´ organs. For example, in T1D one would expect the main changes in the pancreas, while for RA this would be in the joints. The rise of small molecule pharmaceuticals and neurostimulator devices to reduce inflammation is exciting and seems promising for patients with autoimmune diseases since they can be adjusted to the patient needs. Thus, understanding the role of sympathetic nerve-macrophage communication during autoimmune disease development would be helpful in the development of personalized treatments for various autoimmune diseases.
